# Characterization of familial breast cancer in Saudi Arabia

**DOI:** 10.1186/1471-2164-16-S1-S3

**Published:** 2015-01-15

**Authors:** Adnan Merdad, Mamdooh A Gari, Shireen Hussein, Shadi Al-Khayat, Hana Tashkandi, Jaudah Al-Maghrabi, Fatma Al-Thubaiti, Ibtessam R Hussein, Taha Koumosani, Nehad Shaer, Adeel G Chaudhary, Adel M Abuzenadah, Mohammed H Al-Qahtani, Ashraf Dallol

**Affiliations:** 1 Department of Surgery, Faculty of Medicine, King Abdulaziz University, Jeddah, Kingdom of Saudi Arabia; 2 Center of Excellence in Genomic Medicine Research, King Abdulaziz University, Jeddah, Kingdom of Saudi Arabia; 3 Department of Medicine, King Abdulaziz University Hospital, Jeddah, Kingdom of Saudi Arabia; 4 Department of Pathology, King Abdulaziz University Hospital, Jeddah, Kingdom of Saudi Arabia; 5 Department of Biochemistry, Faculty of Science, King Abdulaziz University, Jeddah, Kingdom of Saudi Arabia; 6 KACST Technology Innovation Center in Personalized Medicine, King Abdulaziz University, Jeddah, Kingdom of Saudi Arabia

**Keywords:** hereditary breast cancer, DNA repair, whole-exome sequencing

## Abstract

**Background:**

The contribution of genetic factors to the development of breast cancer in the admixed and consanguineous population of the western region of Saudi Arabia is thought to be significant as the disease is early onset. The current protocols of continuous clinical follow-up of relatives of such patients are costly and cause a burden on the usually over-stretched medical resources. Discovering the significant contribution of BRCA1/2 mutations to breast cancer susceptibility allowed for the design of genetic tests that allows the medical practitioner to focus the care for those who need it most. However, BRCA1/2 mutations do not account for all breast cancer susceptibility genes and there are other genetic factors, known and unknown that may play a role in the development of such disease. The advent of whole-exome sequencing is offering a unique opportunity to identify the breast cancer susceptibility genes in each family of sufferers. The polymorphisms/mutations identified will then allow for personalizing the genetic screening tests accordingly. To this end, we have performed whole-exome sequencing of seven breast cancer patients with positive family history of the disease using the Agilent SureSelect™ Whole-Exome Enrichment kit and sequencing on the SOLiD™ platform.

**Results:**

We have identified several coding single nucleotide variations that were either novel or rare affecting genes controlling DNA repair in the BRCA1/2 pathway.

**Conclusion:**

The disruption of DNA repair pathways is very likely to contribute to breast cancer susceptibility in the Saudi population.

## Background

The discovery of the BRCA1 and BRCA2 genes as major breast cancer susceptibility genes led to great advances in the genetic screening for the disease and the understanding of its inheritance [1,2]. Several other genes were found to play a role in increasing susceptibility to breast cancer but at a markedly lower frequency and penetrance. These genes include ATM, TP53, CHECK2, PTEN, STK11, PALB2, BRIP and the RAD51 genes [3-11]. GWAS studies led to the identification of 21 susceptibility loci that are considered only as low risk alleles [9,12-17]. All these factors combined can account for only 35% of heritable breast cancer with the majority of cases remain with an unknown genetic etiology [18]. This problem is confounded for the admixed and consanguineous population of the western region of Saudi Arabia where virtually no research has been done so far to elucidate the genetic background of heritable breast cancer. A remarkable characteristic of breast cancer in this population is the relatively younger age of onset of the disease where the majority of cases (sporadic or familial) are diagnosed with invasive ductal carcinoma before they are 50 years old [19]. This early onset could be attributed, at least partly, to undetermined genetic susceptibility factors accumulating in the population due to consanguineous marriages and increased exposure to environmental insults due to life-style shifts in the past two decades.

Sanger sequencing of all known breast cancer susceptibility genes could be a daunting task. Developments in massively parallel sequencing technology and whole-exome sequencing alleviate many of the problems associated with such approach and allow for the simultaneous determination of known factors as well as the discovery of novel ones. And in the age of personalized medicine, whole-exome sequencing of each breast cancer patient is fast becoming a standard approach towards genetic diagnosis [20]. In the present study we employed whole-exome sequencing of seven cases diagnosed with familial breast cancer and with unknown BRCA1 or BRCA2 status. We determined the BRCA1 and BRCA2 status in these cases and report the identification of several rare variants that can potentially explain breast cancer susceptibility in each case analyzed.

## Materials and methods

### Patients’ samples

Patients were selected for this study if they have a first-degree relative(s) diagnosed with breast cancer. Peripheral blood was obtained from the patients following obtaining their informed consent and their family history of breast cancer. Patients’ recruitment and blood sampling was all performed according to the institutional ethical procedures (Additional file 1 Figure S1). Genomic DNA was prepared using the Qiagen QIAamp DNA Blood Mini kit according to the manufacturer’s recommendations.

### Whole-exome sequencing and SNP genotyping

Three micrograms of genomic DNA was sheared using the Covaris S2 system. Exome capture was performed on seven cases and six non-cancer controls using the SureSelect Whole-Exome Enrichment version 2 kit from Agilent. Fragment libraries were prepared from the captured exomes for sequencing on the SOLiD 4 platform (AB). Sequencing for each library was performed on one part of the quad slide and fragments were sequenced in in single reads of 50 bp. Sequence capture and primary analysis were performed by the instruments ICS and SETS softwares. SNP genotyping using Taqman was performed using assay ID (C___7530120_20) from Life Technologies targeting the rs1799950 SNP. Genotyping was performed on DNA from peripheral blood of breast cancer patients or non-cancer controls.

### Analysis pipeline

Color-space sequences in .csfasta and .qual files were exported to LifeScope software were mapping to the human genome version 19 (hg19) was performed using standard settings. Identification of single nucleotide polymorphisms was achieved by the diBayes software incorporated in the LifeScope pipeline. Variant call format (vcf) files were analyzed using the SNPs & Variation Suite 7 (SVS7) from Golden Helix where short-listing of candidate SNVs was performed by filtering the detected SNVs to include only those with more than 10x coverage and MQV of >=20. Rare variants were identified by filtering out SNVs present in the 1000genomes or NHLBI Exome sequencing data. Disease-associated SNVs were determined following filtering out rare SNVs found in the 6 non-cancer control cases from the same ethnic background. Damaging nonsynonymous variations were determined by the SIFT, PolyPhen or Mutation Taster softwares within the SVS7 suite.

## Results

Exome sequencing revealed several single nucleotide variants affecting key genes that could be involved in increased susceptibility to breast cancer. The single nucleotide variants or short indels obtained for every sample were filtered against the NHLBI Exome project and the 1000genomes project databases. Novel or rare variants (MAF of <0.01) were filtered against our in-house database of exome sequencing of non-cancer patients or healthy individuals. The statistics of each breast cancer exome sequenced are shown in Table 1. The mutational status of BRCA1 and BRCA2 in the sequenced samples was unknown. Therefore, variants affecting those genes were analyzed first. We have identified one novel frameshift mutation affecting BRCA2 caused by an -/AC insertion affecting one patient only (Table 2). Other BRCA1 or BRCA2 variants identified were previously reported in dbSNP137. However, the nonsense variant represented by SNP rs80358972 is very rare and no information about its MAF could be found. We have found this variant in one BC patient only. Other Missense single nucleotide variants affecting BRCA1 and BRCA2 were identified. However, when selection is based on rarity and degree of predicted damage to the protein, SNP rs1799950 is found in one patient. In order to determine the frequency of the rs1799950 SNP in our cohort, we performed Taqman® SNP genotyping assay on DNA obtained from the peripheral blood of 204 breast cancer patients samples as well as 120 non-cancer controls. The rs1799950 SNP was in a highly significant Hardy-Weinberg disequilibrium in the patient group (X^2^=133.124) compared to the control group (X^2^=0.108). The GG state of the rs1799950 SNP is significantly associated with breast cancer compared to the AA and AG states combined (p=0.0003, OR=22.79, CI=1.366-380.1).

**Table 1:** Next-generation run statistics for breast cancer exomes.

	case_193	case_195	case_264	case_320	case_573	case_574	case_903
Total reads	108,381,083	84,054,514	95,591,607	89,014,487	107,417,081	89,456,100	62,675,973
Mappable reads (%)	73%	67%	72%	73%	78%	64%	78%
Mappable yield (bp)	3,958,042,800	2,795,592,150	3,425,085,950	3,231,704,750	4,145,428,250	2,865,812,200	2,459,039,800
On-target yield (bp) (%)	46%	61%	74%	47%	63%	68%	52%
Coverage of target region (>10x)	71.24	67.68	67.21	64.93	80.59	70.16	64.64
Mean read depth of targeted region	34.63	33.02	45.09	28.08	49.87	37.42	24.14
Mean read depth of called variants	36.54	72.34	68.94	39.41	56.47	44.61	33.35
Number of high quality variants	23959	1788	5823	10019	18691	14113	9623
Number of missense, nonsense, splice, and indel variants	1861	154	352	619	1240	1035	616
Number of filtered damaging rare variants	53	6	21	39	105	91	45
Number of filtered damaging novel variants	141	46	108	80	116	129	71
Number of filtered damaging novel indels	85	38	129	55	54	38	123

**Table 2:** Detection of single nucleotide variations and short indels in the BRCA1 and BRCA2 genes in familial breast cancer cases

Gene	dbSNP137	Genomic Position	AA Change	Nucleotide change	SIFT Prediction	MAF	Occurrence
**BRCA1**	rs799917	Chr17:41244936	p.Pro871Leu	c.2894G>A	Tolerated	0.483	4/7
	rs4986852	Chr17:41244429	p.Ser1040Asn	c.3351 G>A	Tolerated	0.012	1/7
	rs1799966	Chr17:41223094	p.Ser1613Gly	c.5096A>G	Damaging	0.327	3/7
	rs16941	Chr17:41244435	p.Glu1038Gly	c.3345A>G	Damaging	0.303	3/7
	rs1799950	Chr17:41246481	p.Gln356Arg	c.1299A>G	Damaging	0.028	1/7
	rs16942	Chr17:41244000	p.Lys1183Arg	c.3780A>G	Tolerated	0.324	2/7
							
**BRCA2**	rs144848	Chr13:32906729	p.Asn372His	c.1341A>C	Tolerated	0.240	4/7
	rs766173	Chr13:32906480	p.Asn289His	c.1092A>C	Damaging	0.058	1/7
	rs169547	Chr13:32929387	p.Val2466Ala	c.7397C>T	Tolerated	0.022	5/7
	rs80358972	Chr13:32930609	p.Arg2494Stop	c.7707C>T	NA	NA	1/7
	rs4987117	Chr13:32914236	p.Thr1915Met	c.5971C>T	Tolerated	0.011	1/7
	Novel	Chr13:32906700	p.Thr363fs	c.1084_1085insAC	NA	NA	1/7

**Figure 1:**
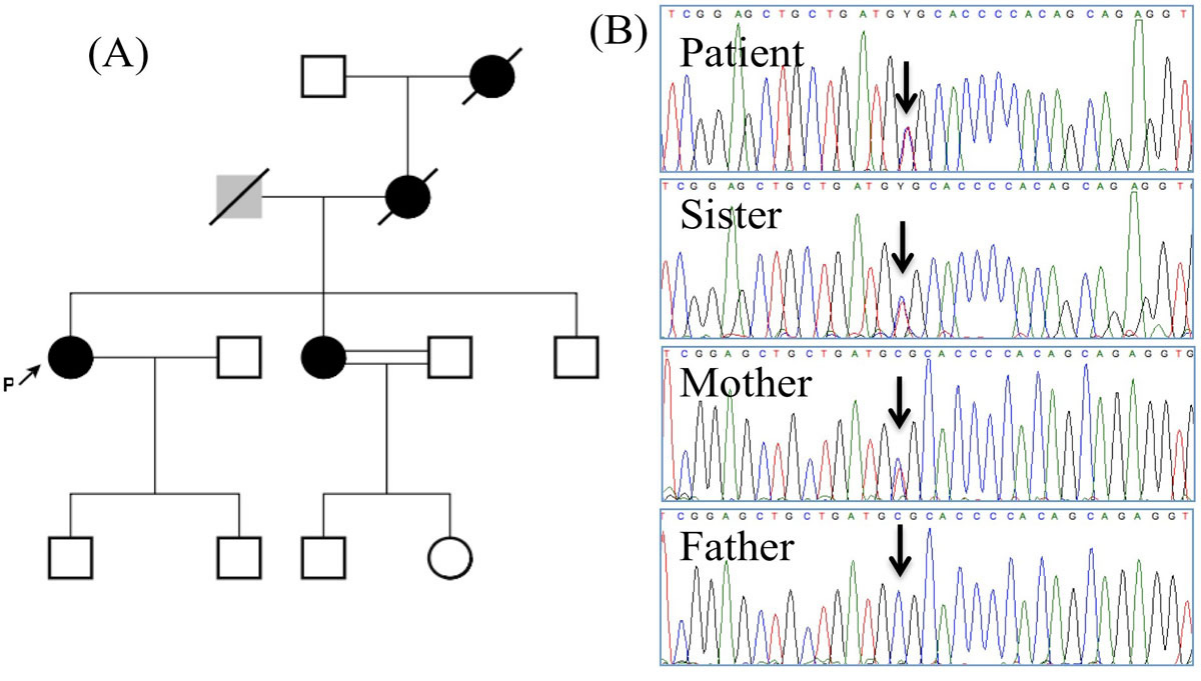
Identification of ERCC6 mutation p.Ala62Thr in a breast cancer family. (A) The pedigree of the family analyzed where the patient, marked with an arrow has a sister who was also diagnosed of breast cancer. The sisters’ mother and grandmother died of breast cancer. Their father died of unspecified lung disease. (B) Sequence chromatographs showing the heterozygous state of the c.148G>A p.Ala62Thr mutation and its segregation in the family. The sequence of the reverese strand is shown.

Predisposition to breast cancer is often caused by genetic defects in DNA repair mechanisms. Therefore, SNVs affecting known genes with DNA repair function were examined (Table 3). In addition, SNVs were also identified affecting the APC, EGF and EGFR genes. An interesting mutation c.148G>A / p.Ala62Thr is found affecting the PARP1-interacting region of the Cockayne Syndrome group B (ERCC6) gene. Analysis of DNA from the family of the affected female revealed that this mutation segregated in the heterozygous state in one sibling affected with breast cancer as well as in the mother who also suffered from breast cancer. The father did not harbor this mutation (Figure 1). This SNV was recently reported by the 1000Genome project (rs186839348) where it was found only once in 1094 individuals. We could not detect this SNV in 228 non-cancer control samples from Saudi Arabia.

**Table 3:** Candidate breast cancer genetic risk factors

Chr.	Position	Gene Name	Refseq	Nucleotide change	Amino acid change
9	133748391	ABL1	NM_007313	c.1109T>C	p.Met370Thr
4	41015755	APBB2	NM_001166050	c.680C>G	p.Ser227*
5	112157653	APC	NM_001127511	c.1319T>C	p.Phe440Ser
5	112175675	APC	NM_001127511	c.4330A>G	p.Lys1444Glu
X	55028750	APEX2	NM_014481	c.308T>G	p.Phe103Cys
2	68740731	APLF	NM_173545	c.541G>A	p.Glu181Lys
9	32987778	APTX	NM_001195249	c.247C>A	p.Pro83Thr
7	97498324	ASNS	NM_133436	c.145C>T	p.Arg49Trp
3	48506356	ATRIP	NM_032166	c.2101C>T	p.His701Tyr
12	56994493	BAZ2A	NM_013449	c.4580G>A	p.Arg1527Gln
11	117261519	CEP164	NM_014956	c.1961C>T	p.Ala654Val
11	72070023	CLPB	NM_030813	c.766A>C	p.Asn256His
11	61097050	DDB1	NM_001923	c.334A>T	p.Ile112Phe
11	61099086	DDB1	NM_001923	c.139G>A	p.Glu47Lys
1	10529353	DFFA	NM_004401	c.179C>T	p.Thr60Ile
11	46396161	DGKZ	NM_201532	c.1517T>C	p.Phe506Ser
1	44680377	DMAP1	NM_001034023	c.200A>T	p.Asp67Val
4	110901232	EGF	NM_001178130	c.2158C>T	p.Arg720Cys
4	110915959	EGF	NM_001178130	c.2805T>A	p.Cys935*
7	55231449	EGFR	NM_201282	c.1655A>C	p.Asn552Thr
10	50740827	ERCC6	NM_000124	c.184G>A	p.Ala62Thr
16	89851328	FANCA	NM_000135	c.1404G>T	p.Lys468Asn
6	30521271	GNL1	NM_005275	c.664C>G	p.Leu222Val
10	96306191	HELLS	NM_018063	c.89T>A	p.Met30Lys
1	153742705	INTS3	NM_023015	c.2421G>C	p.Gln807His
1	153744835	INTS3	NM_023015	c.2740A>T	p.Lys914*
6	42985075	KLHDC3	NM_057161	c.145T>G	p.Phe49Val
17	8273384	KRBA2	NM_213597	c.547C>T	p.Arg183*
3	49167350	LAMB2	NM_002292	c.1327C>T	p.His443Tyr
6	52129538	MCM3	NM_002388	c.2275C>A	p.His759Asn
6	52148114	MCM3	NM_002388	c.169A>T	p.Lys57*
7	99691911	MCM7	NM_182776	c.1205C>A	p.Ala402Asp
6	30675438	MDC1	NM_014641	c.2918C>G	p.Ala973Gly
12	68719303	MDM1	NM_017440	c.551A>C	p.Asn184Thr
1	46073578	NASP	NM_001195193	c.803A>C	p.Gln268Pro
13	25049687	PARP4	NM_006437	c.1837C>T	p.Leu613Phe
22	38461037	PICK1	NM_001039583	c.182A>C	p.Asp61Ala
12	133237560	POLE	NM_006231	c.3055A>G	p.Ser1019Gly
12	133240651	POLE	NM_006231	c.2645A>G	p.Asn882Ser
6	43550079	POLH	NM_006502	c.23T>G	p.Val8Gly
11	7660974	PPFIBP2	NM_003621	c.1248C>A	p.Phe416Leu
11	7670122	PPFIBP2	NM_003621	c.1889T>C	p.Leu630Pro
2	1670120	PXDN	NM_012293	c.1157A>T	p.Asp386Val
2	1680732	PXDN	NM_012293	c.815A>T	p.Asn272Ile
19	13063505	RAD23A	NM_005053	c.816A>T	p.Gln272His
12	110957646	RAD9B	NM_152442	c.815T>A	p.Ile272Asn
4	39310618	RFC1	NM_001204747	c.1523A>C	p.Gln508Pro
9	94486015	ROR2	NM_004560	c.2761C>T	p.Pro921Ser
9	135171409	SETX	NM_015046	c.5956A>T	p.Arg1986Trp
9	135205531	SETX	NM_015046	c.1454G>C	p.Trp485Ser
6	146244812	SHPRH	NM_001042683	c.3512A>G	p.Lys1171Arg
10	69672638	SIRT1	NM_012238	c.1765G>T	p.Glu589*
16	18846451	SMG1	NM_015092	c.8093C>A	p.Thr2698Lys
12	104376700	TDG	NM_003211	c.602A>C	p.Lys201Thr
14	24711133	TINF2	NM_001099274	c.260T>C	p.Phe87Ser
17	38546386	TOP2A	NM_001067	c.4298A>C	p.Lys1433Thr
17	18178184	TOP3A	NM_004618	c.2938A>T	p.Lys980*
18	662218	TYMS	NM_001071	c.352T>G	p.Leu118Val
15	70971987	UACA	NM_001008224	c.812T>C	p.Val271Ala
8	103324043	UBR5	NM_015902	c.2338C>A	p.Gln780Lys
8	103359274	UBR5	NM_015902	c.433G>T	p.Gly145Cys
8	30949362	WRN	NM_000553	c.1846G>C	p.Ala616Pro
8	31030529	WRN	NM_000553	c.4210A>G	p.Lys1404Glu
12	58345668	XRCC6BP1	NM_033276	c.443C>T	p.Ala148Val

## Discussion

Breast cancer incidence is on the rise in the Kingdom of Saudi Arabia with a remarkable number of those affected are being diagnosed before they are 50 years old [19]. The early-onset of the breast cancer in this population could be partly explained by the accumulation of breast cancer predisposition genetic factor(s) due to high incidence of consanguineous marriages. The effects of these genetic factor(s) is probably becoming more evident now due to the social and life-style changes brought upon by the relatively recent positive economical upheavals in the country. In order to identify such genetic factors, we performed a pilot whole-exome sequencing study on DNA obtained from the peripheral blood of seven cases suffering from hereditary breast cancer. First, the status of the known breast cancer predisposition factors, mainly BRCA1 and BRCA2, was determined. We could not identify recurrent BRCA1/2 mutations in our cohort. However, we identified a novel insertion that led to a frameshift mutation (p.Thr363fs) in BRCA2 causing the synthesis of a truncated and presumably dysfunctional protein. We identified another rare mutation in BRCA2 in one of our patients. Represented by the rs80358972 SNP, the p.Arg2494Stop affecting BRCA2 has been reported by the Breast Cancer Information Core submitted by Myriad Genetics as a direct result of their diagnostic services. Additionally, we have identified the relatively rare rs1799950 SNP in BRCA1 which is a p.Gln356Arg mutation reported by the 1000Genomes project to have an MAF of 0.026. We found this SNP in our cohort with a MAF of 0.058 (7 heterozygous cases in 120 non-cancer cases). The minor allele frequency of the rs1799950 SNP did not differ significantly from controls. However, we observed an increase in the number of breast cancer cases displaying the homozygous GG minor allele state that is not seen in the control cases. When the GG state is analyzed in comparison to the combined frequency of the AA and AG states, a highly significant association with breast cancer becomes evident. The rs1799950 SNP is one of 25 SNPs in cancer predisposition genes that were identified to confer minor but cumulatively significant risk of breast cancer [21]. However, a later study dismissed the association of the rs1799950 SNP with breast cancer [22]. Unfortunately, it is difficult to perform direct comparisons between our findings and reported studies due to the differences in sample size and the ethnic makeup of the cohorts analyzed.

Whole-exome sequencing revealed several candidate risk factors for breast cancer. We made the assumption that the most likely risk factor is a gene(s) involved in DNA repair, cell cycle or apoptosis [18]. Applying this filter to the SNVs obtained reveal rare polymorphisms that could affect important genes such as WRN, APC, EGF, EGFR and ERCC6. The contribution of these SNVs towards increasing predisposition to breast cancer remains unknown. Therefore, we analyzed the segregation with breast cancer of the SNVs affecting ERCC6 (p.Ala62Thr) and WRN (p.Ala616Pro) in a family with reported breast cancer affecting three generations (case_574). The WRN p.Ala616Pro was detectable in the two siblings diagnosed with breast cancer. However, this SNV could not be found in the mother who died of breast cancer. In contrast, the ERCC6 p.Ala62Thr SNV segregated with breast cancer in the same family and it was not detectable in the father or control samples. This mutation affects the PARP1-interaction region of ERCC6, also known as Cockayne Syndrome group B (CSB) [23]. ERCC6-dependent activation of the poly(ADP-ribose)polymerases, or PARPs is an early event in the cellular response to genotoxic stress [24]. Carrying a variant ERCC6 therefore will cause a less-efficient DNA repair response and could therefore lead to an increased predisposition to breast cancer.

## Conclusions

This is the first report on the breast cancer predisposition factors in the population of the Kingdom of Saudi Arabia. The high consanguinity and life-style shifts in this population are coupled to an early-onset breast cancer and the SNVs identified in this study could partly explain this phenomenon. We have identified a novel BRCA2 mutation as well as found a case with a very rare nonsense mutation truncating the BRCA2 protein. We demonstrate the potential importance of the homozygous risk allele to breast cancer predisposition in the Saudi population. We suggest that mutations in the ERCC6 gene could be considered as potential risk factors for breast cancer. Although no recurrent mutations were identified, this study validates the use of whole-exome sequencing for the determination of the “breast cancer predisposition genome”.

## Competing interests

The authors declare that they have no competing interests.

## Authors’ contributions

AM conceived the study, collected samples and interviewed patients. MG collected samples and contributed to the study design. SH performed the whole-exome analysis and Sanger sequencing. SK supported patients’ recruitment to the study. HT and FT contributed to patients’ recruitment. JM provided archival samples from patients’ relatives. IRH interviewed the patients and provided genetic counseling. TK, AGC, AMA and MHQ contributed to the study design. NS performed whole-exome sequencing, AD developed the study, performed whole-exome sequencing and bioinformatics and wrote the manuscript.

## Supplementary Material

Additional file 1 **Figure S1** Pedigrees showing the inheritance pattern of breast cancer in the families recruited to this study. Dark filled circles represent cases with breast cancer which is indicated with a small arrow if included in the exome sequencing. Click here for file

## Declarations

Publication charges for this article have been funded by the Center of Excellence in Genomic Medicine Research, King Abdulaziz University

This article has been published as part of *BMC Genomics* Volume 16 Supplement 1, 2015: Selected articles from the 2nd International Genomic Medical Conference (IGMC 2013): Genomics. The full contents of the supplement are available online at http://www.biomedcentral.com/bmcgenomics/supplements/16/S1.
