# Supplementary figures and images for: Characterization of familial breast cancer in Saudi Arabia
Source: BMC Genomics. 2015 Jan 15;16(Suppl 1):S3. doi: 10.1186/1471-2164-16-S1-S3 (PMC4315159; doi:10.1186/1471-2164-16-S1-S3)

Figure S1

Family 195

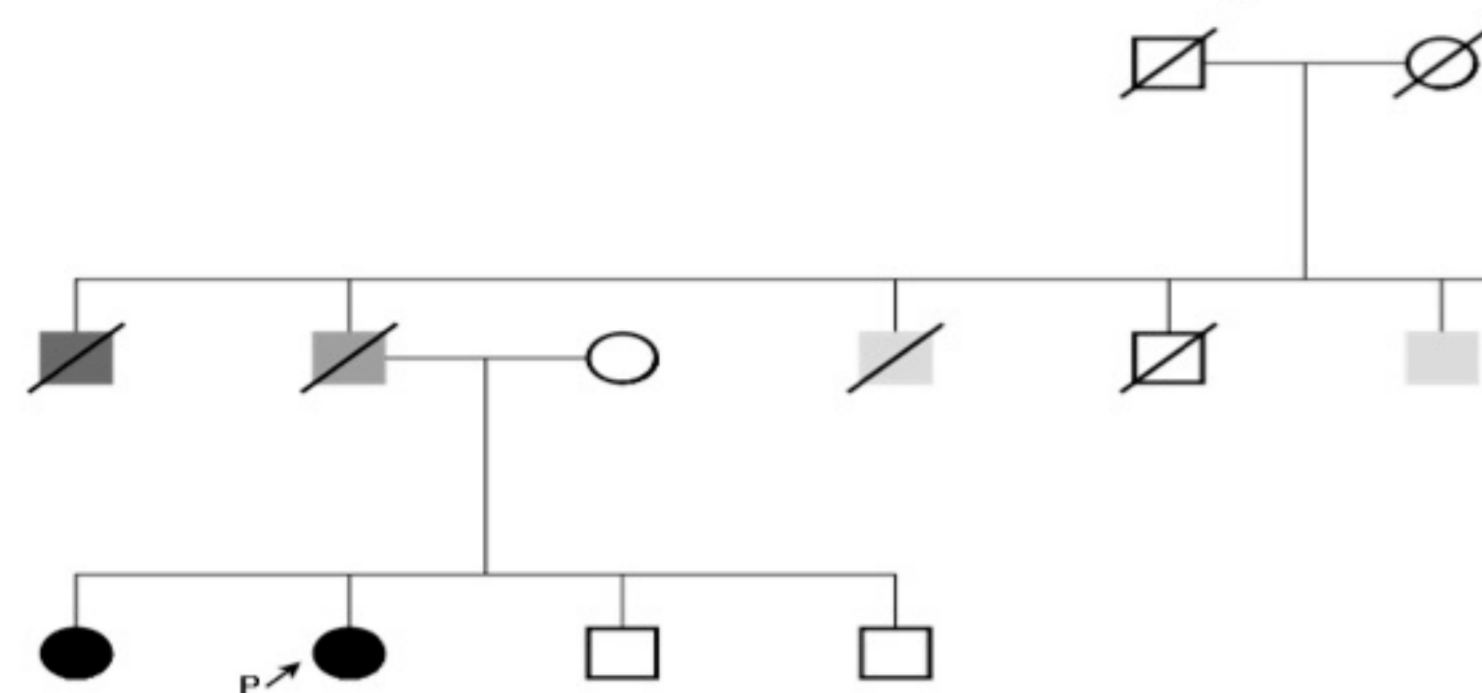

Family 193

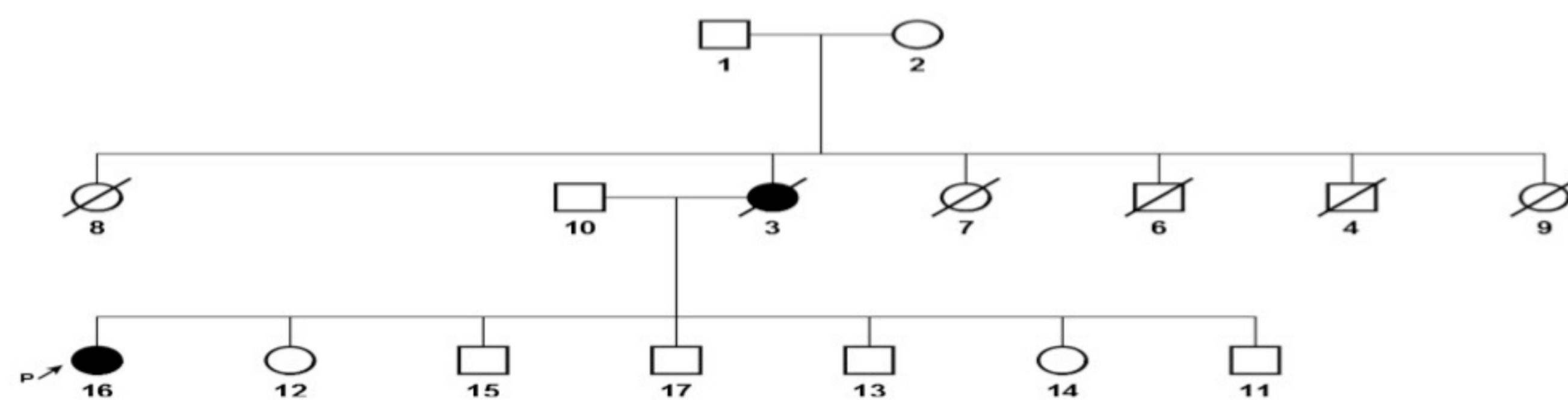

Family 264

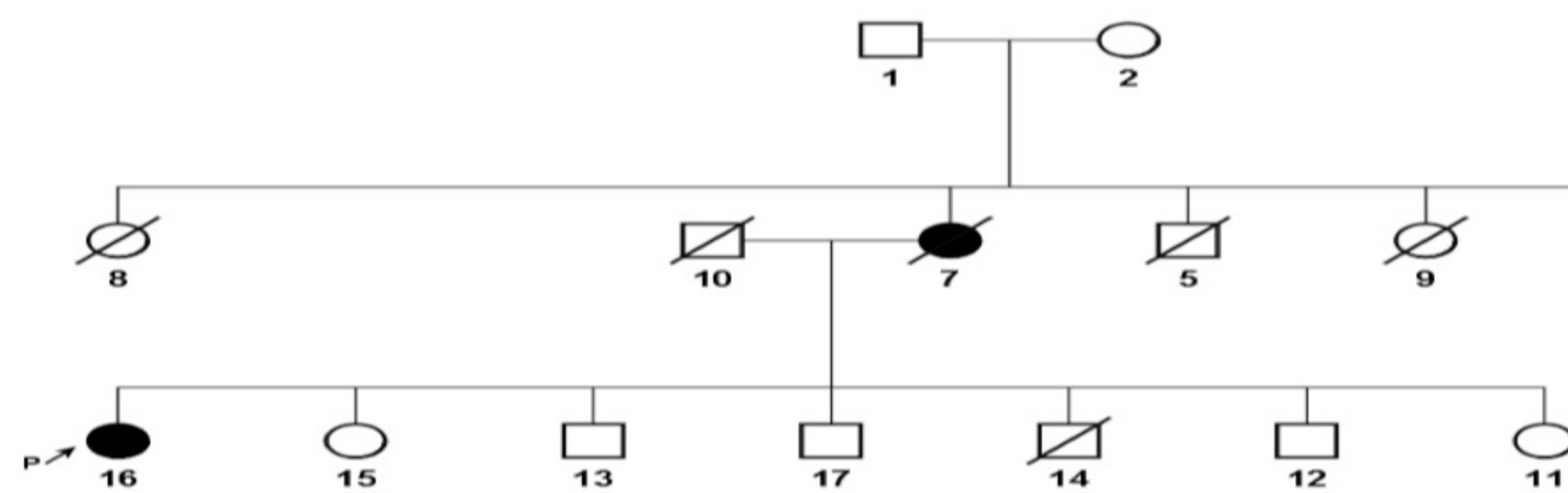

Family 320

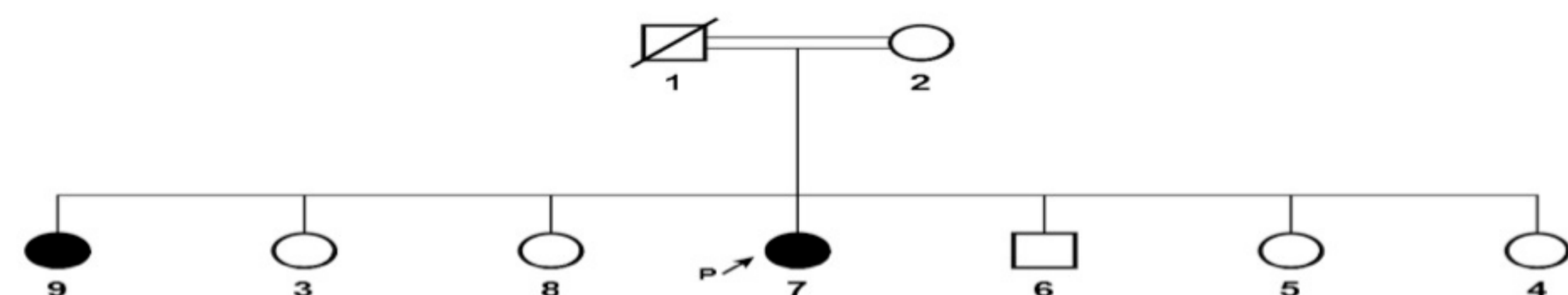

Family 573

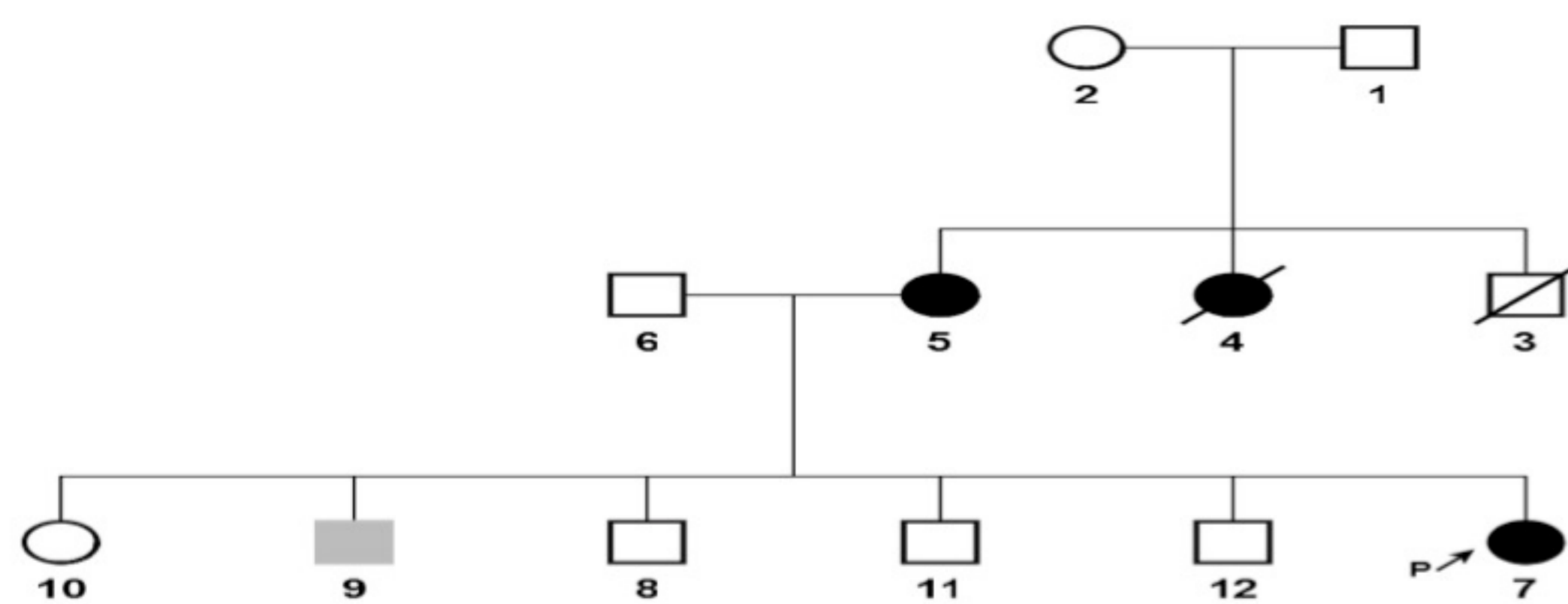

Family 574

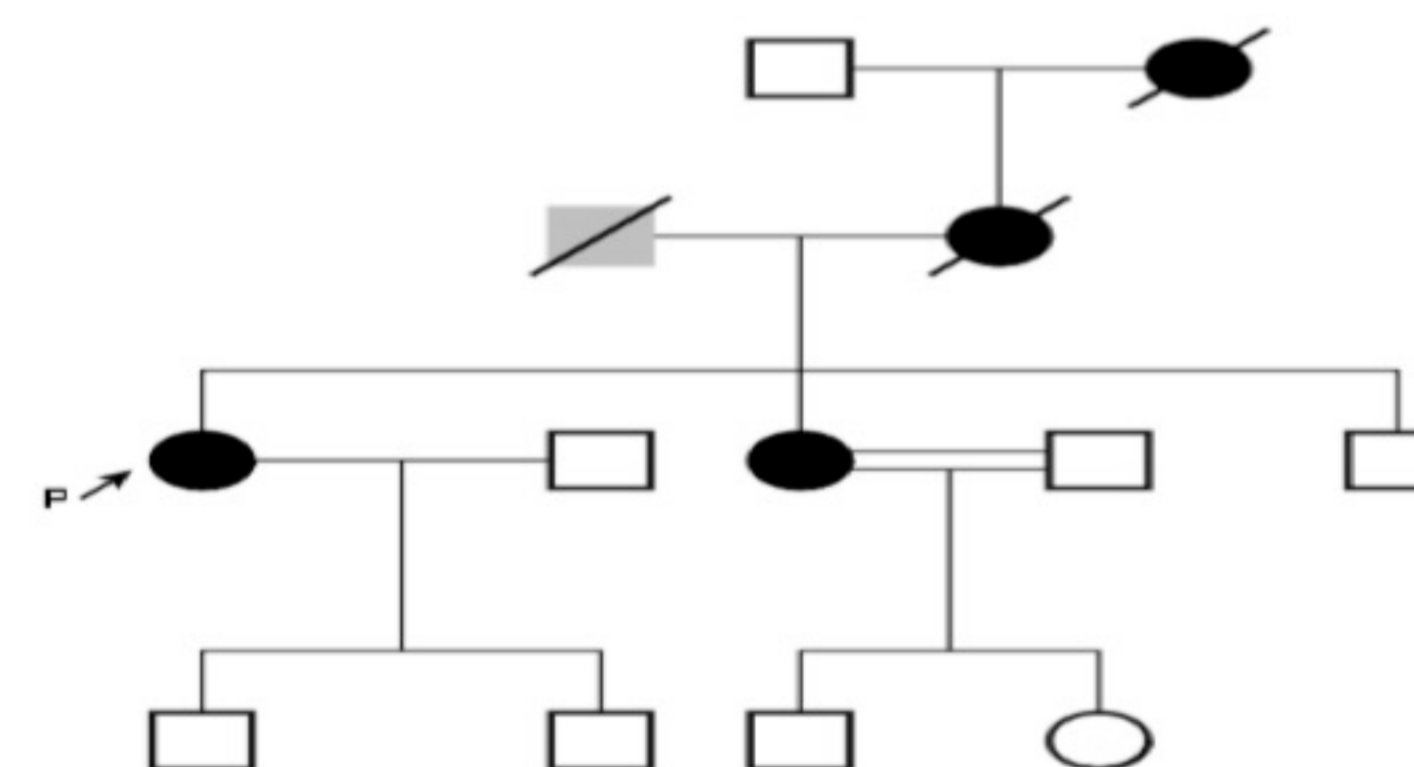

Supplement: Additional file 1 — Figure S1 Pedigrees showing the inheritance pattern of breast cancer in the families recruited to this study. Dark filled circles represent cases with breast cancer which is indicated with a small arrow if included in the exome sequencing. [file 1471-2164-16-S1-S3-S1.pdf]
